# Droplet microreactor for high-throughput fluorescence-based measurements of single catalyst particle acidity

**DOI:** 10.1038/s41378-023-00495-2

**Published:** 2023-03-30

**Authors:** Jeroen C. Vollenbroek, Anne-Eva Nieuwelink, Johan G. Bomer, Roald M. Tiggelaar, Albert van den Berg, Bert M. Weckhuysen, Mathieu Odijk

**Affiliations:** 1 BIOS Lab on a Chip Group, MESA+ Institute, University of Twente, Hallenweg 15, 7522 NH Enschede, The Netherlands; 2 Inorganic Chemistry and Catalysis, Debye Institute for Nanomaterials Science, Utrecht University, Universiteitsweg 99, 3584 CG Utrecht, The Netherlands; 3 NanoLab Cleanroom, MESA+ Institute, University of Twente, Hallenweg 15, 7522 NH Enschede, The Netherlands

**Keywords:** Chemistry, Engineering

## Abstract

The particles of heterogeneous catalysts differ greatly in size, morphology, and most importantly, in activity. Studying these catalyst particles in batch typically results in ensemble averages, without any information at the level of individual catalyst particles. To date, the study of individual catalyst particles has been rewarding but is still rather slow and often cumbersome^1^. Furthermore, these valuable in-depth studies at the single particle level lack statistical relevance. Here, we report the development of a droplet microreactor for high-throughput fluorescence-based measurements of the acidities of individual particles in fluid catalytic cracking (FCC) equilibrium catalysts (ECAT). This method combines systematic screening of single catalyst particles with statistical relevance. An oligomerization reaction of 4-methoxystyrene, catalyzed by the Brønsted acid sites inside the zeolite domains of the ECAT particles, was performed on-chip at 95 °C. The fluorescence signal generated by the reaction products inside the ECAT particles was detected near the outlet of the microreactor. The high-throughput acidity screening platform was capable of detecting ~1000 catalyst particles at a rate of 1 catalyst particle every 2.4 s. The number of detected catalyst particles was representative of the overall catalyst particle population with a confidence level of 95%. The measured fluorescence intensities showed a clear acidity distribution among the catalyst particles, with the majority (96.1%) showing acidity levels belonging to old, deactivated catalyst particles and a minority (3.9%) exhibiting high acidity levels. The latter are potentially of high interest, as they reveal interesting new physicochemical properties indicating why the particles were still highly acidic and reactive.

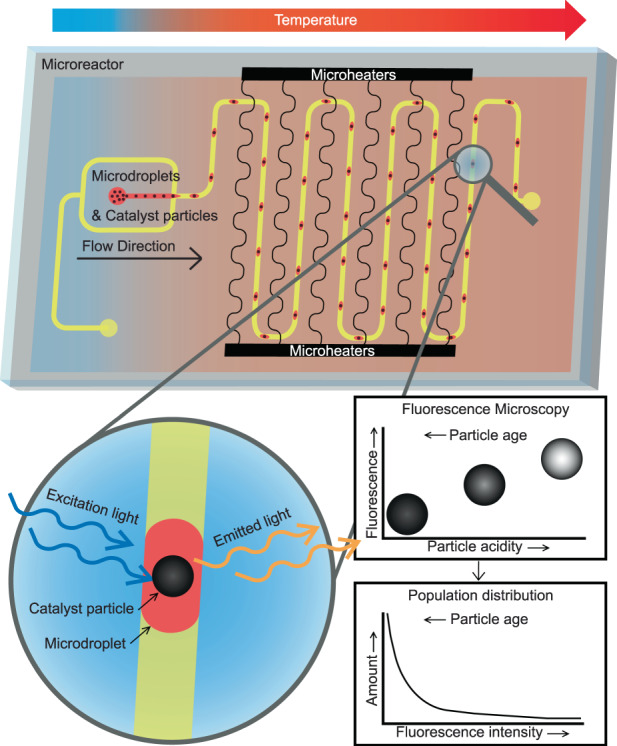

## Introduction

Fluid catalytic cracking (FCC) is an essential process for the production of gasoline in which vacuum gas oil (VGO) is cracked after distillation. The technique is currently studied for its potential to crack municipal and agricultural wastes, including plastic wastes, thereby contributing to a more circular society^2^. The FCC process makes use of a 50–150 µm multicomponent heterogeneous catalyst consisting of a zeolite, matrix, filler, and binder. Among these components, the Brønsted acid sites in the zeolite domains are often considered the main active components^3–5^. The catalyst particles are transported through a riser reactor in a few seconds and behave like a fluidized bed, hence the name fluid catalytic cracking. After the reaction, the products are separated from the catalyst, and consecutively, the catalyst particles are rejuvenated by stripping off the residual products and coke by heating them in an oxygen-rich environment in a regenerator reactor. Thereafter, the rejuvenated catalyst particles continue their cycle in the riser reactor^3^. In every cycle, catalyst particles are deactivated by the harsh FCC reaction conditions. This irreversible process is due to dealumination of the zeolite domains and the accumulation of metals inside the porous structure of the particles^3,6^. To prevent a gradual loss of activity, a small fraction of the spent FCC catalyst is constantly replaced with fresh catalyst particles. The result is a catalyst mixture with an age distribution, the so-called equilibrium catalyst (ECAT).

Bulk and single particle characterization methods have been used to better understand the loss of activity seen for the spent catalyst. The bulk approach enables the study of large batches of catalyst particles, providing bulk statistics at the cost of averaging out single particle heterogeneities. The single particle approach provides many details on the single catalyst particles with losses of speed and statistical relevance. However, for systems with large interparticle heterogeneities, such as the ECAT used industrially, neither bulk measurements that average out the characteristics of many particles on the one hand nor single particle analyses without statistical relevance on the other hand are sufficient to fully reveal the composition of the particle population. This does not imply that either analytical approach is not valuable. Bulk measurements are, for example, very useful for analyses of the physicochemical properties of ECATs, such as acidity, pore accessibility, and the degree of metal poisoning^1,3^. The analyses of single catalyst particles provide valuable in-depth research results elucidating the deactivation processes of FCC particles. The deposition of metals, such as iron (Fe) and nickel (Ni), as well as changes in pore structure, acidity, and accessibility, have been reported with high spatial resolution, even down to the nanometer scale, thanks to the use of X-ray microscopy (XRM) as well as single-molecule fluorescence (SMF) microscopy^7,8^. It is of course very important to gain this in-depth information^1^, but it is also very time-consuming to perform all of these single catalyst particle measurements. Therefore, these extensive analyses have only been performed with a handful of ECAT particles, hampering the statistical relevance of the characterization methods employed.

In an industrial FCC reactor, a shift in the acidity equilibrium between fresh, acidic/active catalyst particles and old, deactivated catalyst particles has a significant influence on the overall performance of the reactor system^3^. A method for rapidly gaining information on the overall acidity ratio of the reactor with a statistically relevant and representative sample would therefore be of great value. Density sorting is commonly used as a means of sorting the most deactivated from the least deactivated catalyst particles, by using the accumulation of metals as a measure for catalytic age. However, the acidity of a catalyst particle is not always related to its density^9^. By using microfluidics for high-throughput analyses of catalyst particles, the level of detail for bulk characterization and the statistical relevance of single particle characterization can be improved. To realize a compromise between the two existing analytical approaches, a high-throughput platform for screening the acidities of single catalyst particles has been developed. This complementary method can be used to perform initial screening of a particle population, after which a follow-up in-depth characterization will provide higher statistical relevance. A microreactor was fabricated in which an emulsion is generated with microfluidic droplets that encapsulate the individual catalyst particles, enabling rapid screening in an easy fashion. Screening with the developed microreactor not only provides information on acidity at the single particle level but also gives a statistical value to these measurements. In recent work, we were able to sort pre-stained catalyst particles from an ECAT mixture based on their individual acidity^10^. It would be extremely beneficial if the staining step could also be performed in the microreactor to guarantee identical reaction conditions for the catalyst particles.

In the field of microfluidics, the use of microdroplets is common practice^11^. Microdroplets are used in many applications, such as for performing chemical reactions to study reaction kinetics, to form products inside the droplets, as traps for single particles or cells, and for biological assays of cells and DNA in droplets^12–15^. By mixing two immiscible fluids such as oily (nonpolar) and watery (polar) substances, either oil-in-water (O/W) or water-in-oil (W/O) droplets are created^12,13,16^. It is crucial for stable droplet formation that the walls of the microfluidic channels, which can be polar or nonpolar, are preferentially wetted by the continuous phase, as demonstrated by Shui et al. ^16^. A droplet is formed as the result of the shear forces and interfacial tension between the two fluids, which creates a stream of the polar liquid (continuous phase) with droplets of the nonpolar liquid (dispersed phase) in the case of polar channel walls^13^. Due to the shear forces acting on the droplets, there is a circulating flow that enhances mixing inside the droplet^12^. Another advantage of using droplet microfluidics instead of continuous flow reactors is that compartmentalizing the analytes of interest effectively precludes dilution by preventing Taylor dispersion^17^, a common issue that can deteriorate the temporal resolution of, e.g., a chromatographic separation^18^ or sampling of neurotransmitters with dialysis probes^19^. Therefore, droplets form ideal reaction environments due to their well-controlled properties such as shape, size, and mono-dispersity. Because of these properties and their volumes ranging from the µL to fL scale, droplets have been referred to as micro- or nanoreactors in the literature^11,20^. Both the configuration of the channels and the flow rates used for both phases are involved in the creation of droplets. Examples of the geometry types formed for on-chip droplet generation are the T-junction and the flow focusing junction^13^. The features of droplet generation, e.g., the squeezing, dripping, and jetting regimes^13^, can be tuned with the flow rate. Typically, increasing the flow rate of the continuous phase relative to that of the dispersed phase results in smaller droplets. Since the microreactor presented in this work is intended for use at elevated temperatures and on-chip droplet formation will be controlled with a flow focusing geometry, the viscosities of the liquids will change during operation. This could potentially change the droplet formation regime as well as affect the Capillary number^12^. Modeling of droplet formation with a flow focusing and T-junction geometry has been performed by Mamet et al.^21^ and van der Graaf et al.^22^, respectively. This provided more details on the physics involved.

To obtain the elevated temperature ranges that some chemical reactions require, a microreactor can either be heated externally or internally. External heating involves producing heat outside the microreactor with, for example, Peltier elements^23^, externally heated fluids^24^, or by placing the complete system in an oil bath^25^. Integrated heating techniques include the use of endo- and exothermic processes^26^, microwave heating^27–29^, and Joule heating^30–34^. Joule heating has the advantages of fast temperature cycling, steep gradients, and localized heating. By locating thin-film metallic structures in close proximity to the microfluidic channels, the channels can be heated efficiently via Joule heating by applying a voltage across these metal microstructures^34–42^. Similar thin-film microheaters have been widely reported in the literature, and their uses vary from biological assays, such as PCR^35–37^, to microreactors for single phase purposes^40,41,43^, as well as to reactions inside droplets in a microreactor^25,44^. When these thin-film structures are created out of platinum, the heaters are stable up to at least 500 °C^40,42^, provided that an appropriate metal adhesion layer is used. Furthermore, metallic thin-film structures can also be used to measure the temperature. Dedicated thin-film temperature sensor structures comprising platinum^42^, nickel^45^, or gold^46^ can be used to measure the temperature of a microfluidic device by exploiting the temperature-dependent resistance of the metal used, and these sensors are known for their high stability and sensitivity. This type of sensor is therefore called a resistance temperature detector (RTD). By monitoring the resistance of these structures, temperature readouts can be achieved in an accurate, reproducible, and fast manner. Measuring the temperature close to the heater provides a feedback mechanism enabling temperature control in the microreactor^40,41,47^. For the droplet microreactor in this work, integrated thin-film platinum structures were used for both heating and temperature read-out.

By using a microfluidic system with integrated metallic thin-film microheaters and temperature sensors^34,39–41,47,48^, the reaction conditions can be tuned and screened rapidly. Microfluidic devices are already used for single-cell analyses with high-throughput rates^12–15^. Since the catalyst particles to be investigated are slightly larger than cells (9x on average), the same techniques can be used for high-throughput analyses of single catalyst particles. However, the higher density of the FCC catalyst particles does provide some challenges that require special care in designing the entire setup, which includes a microfluidic device, tubing, and pumps, making it far from trivial to introduce inorganic particles into the microreactor. Nonetheless, by using droplets as unique nanoreactors^11,16,17,20^, single FCC ECAT particles can be encapsulated in the droplets and screened for their properties, such as activity. As the catalytic activity of FCC particles is linked to the availability of (Brønsted) acid sites in the zeolite domains, the activity of FCC particles can be studied with probe molecules such as thiophene, furfuryl alcohol, and styrene^4,49^. These molecules form fluorescent oligomers after they react with the Brønsted acid sites in the zeolite domains of the FCC particles, which can be easily detected with (confocal) fluorescence microscopy (CFM)^50^ and UV‒vis microspectroscopy^4^. More specifically, the oligomerization reactions of styrene derivatives, such as 4-methoxystyrene, are acid-catalyzed and occur at temperatures between 100 and 200 °C^5^. With the integrated metallic structures used for heating and sensing in the developed droplet microreactor, these temperatures can be reached and controlled. In previous work from Buurmans et al., the previously mentioned microspectroscopic optical techniques were used to investigate the acidity of industrial ECAT particles, and FCC particles deactivated with various laboratory-based deactivation methods, using various styrene-like probe molecules^1,5^. Furthermore, they showed that it is possible to distinguish zeolite USY-based FCC particles from zeolite ZSM-5-based FCC particles. Zeolite USY and ZSM-5 have different framework structures. Due to the differences in micropore sizes and architectures, different styrene oligomers are formed inside these micropores during chemical staining. These differences lead to different colors (and thus different UV‒vis absorption peaks) for the FCC particles with the USY or ZSM-5 zeolites^51^.

**Fig. 1:**
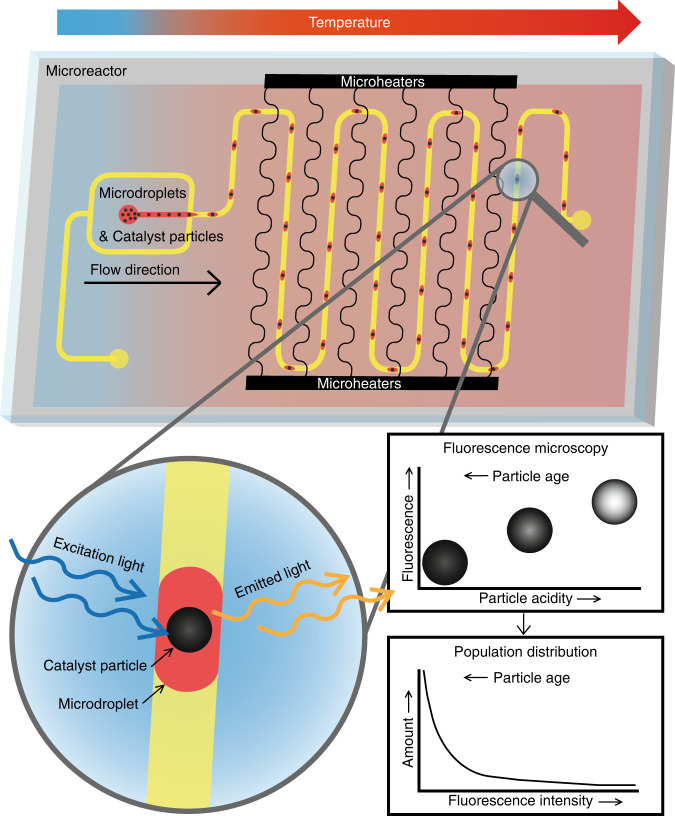
Conceptual representation of the droplet microreactor used for performing reactions in the encapsulated catalyst particles at elevated temperatures. Fluorescence microscopy can be used to screen fluorescent product formation in the catalyst particles. The expected relationships between fluorescence intensity and catalyst particle acidity and age are shown, as is a population distribution of the catalyst particles

In this paper, we present a microfluidic platform dedicated to single catalyst particle diagnostics. FCC ECAT particles are captured in paraffin oil droplets and flushed through the microreactor at 95 °C. A probe molecule, 4-methoxystyrene, is present inside these catalyst particles, as described in the Methods section, and oligomerizes to form a fluorescent reaction product. As validated by previous work from the group, the availability of active sites in the particle is linked to the intensity of the fluorescence signal produced^4^. The fluorescence intensity is measured on-chip for all passing particles through an optical window near the outlet. A conceptual representation of particle encapsulation, on-chip heating with the microheaters, and subsequent fluorescence detection is shown in Fig. 1.

## Results and discussion

### Droplet microreactor design and fabrication

**Fig. 2:**
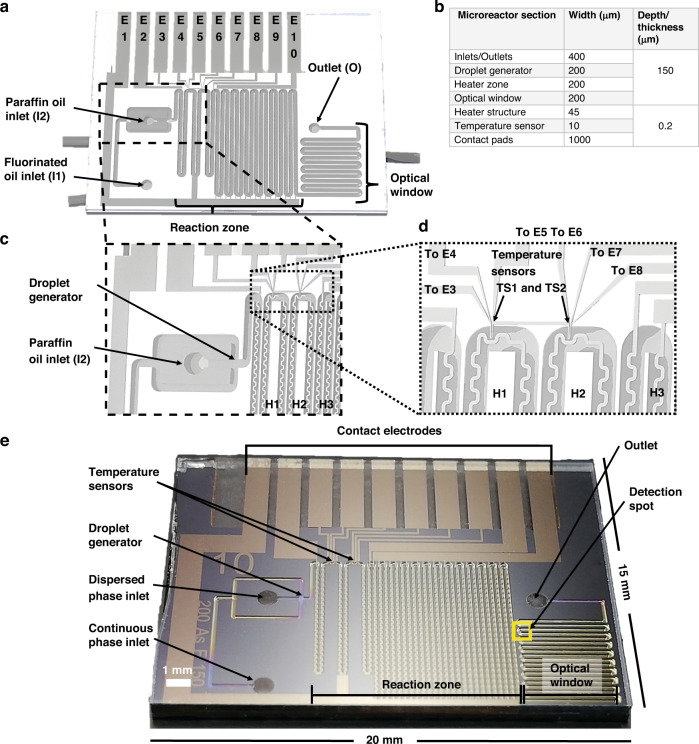
Overview of the droplet microreactor. The channels have widths of 200 µm and depths of 150 µm, the heaters are 45 µm wide and 200 nm thick, and the temperature sensors have widths of 10 µm and thicknesses of 200 nm. All metallic thin-film structures were made from 190 nm platinum (Pt), with a 10 nm tantalum (Ta) adhesion layer. The bottom substrate is silicon (Si) and the top substrate is glass. This figure provides **a** an overview showing the contact electrodes (E1–E10) for the heater and sensor structures, the flow channel layout, the inlets for the dispersed phase (I1) and continuous phase (I2), and the outlet (O); **b** the dimensions of the various sections of the droplet microreactor, **c** a close-up schematic of the droplet generator, showing the flow focusing junction and the inlet for the dispersed phase and the three heaters (H1–H3) that are controlled via electrodes E2 (H1), E9 (H2), and E10 (H3), **d** a close-up schematic of the temperature sensor structures (TS1 and TS2) and the electrodes used for addressing those structures (E3–E8), and **e** the droplet microreactor used for the experiments, including the area for fluorescence detection (indicated by the yellow square)

The design of the droplet microreactor, which has been reported previously^52,53^, is illustrated in Fig. 2a. It features a droplet generator (Fig. 2c), a heated reaction zone, a temperature sensor section (Fig. 2d) and an optical window for fluorescence detection (Fig. 2a). Paraffin oil flows into the microreactor via inlet I2, and fluorinated oil flows via inlet I1. The flow-focusing junction creates paraffin oil-in-fluorinated oil droplets. The heater section contains three separate heaters. Heater 1 (H1) and heater 2 (H2) are controlled separately via electrodes E9 and E2, respectively. Both heaters have interwoven temperature sensors, with which they can be monitored and controlled via LabVIEW software. These temperature sensors are shown in Fig. 2d. A four-point measurement, in which a current is applied to electrodes E3 and E8 and induces a voltage drop over the narrow sensing structure, is used to measure the resistance of the sensor. This voltage can be measured between electrodes E4 and E5 for temperature sensor 1 (TS1) and electrodes E6 and E7 for temperature sensor 2 (TS2). When the temperature increases, the resistance of the sensor increases, resulting in an increasing voltage across the specified electrodes. The final heater section, heater 3 (H3), is a block of parallel thin-film wires. This heater block does not have a dedicated temperature sensor. The parallel section is connected to electrode E10; E1 is the ground electrode to which all heaters are connected. The parallel heater section, as well as the meandering flow channel, is required to allow sufficient residence times at elevated temperatures for chemical reactions to occur. The optical window is used for measuring fluorescence from the droplets, although the heater is narrow enough to monitor the droplets underneath the heaters as well. Figure 2e shows a photo of the microreactor, which has overall dimensions of 15 mm × 20 mm × 1 mm. The microreactors were fabricated in the cleanroom of the MESA + NanoLab at the University of Twente. The channels are 150 µm deep, and the width is 200 µm. These cross-sectional dimensions were chosen based on the average diameter, ca. 75 µm, of the catalyst particles. The heater and temperature sensor structures have widths of 45 µm and 10 µm, respectively. The thickness of the thin-film metal structure is 200 nm (190 nm platinum with a 10 nm tantalum adhesion layer), and is embedded in 200 nm-deep recesses etched into the glass substrate. More details on fabrication of the silicon/glass microreactor can be found in Section SI.1 of the Supplementary Information (SI).

### Droplet microreactor setup and oligomerization reaction

An image of the droplet microreactor used for these experiments involving high-throughput measurements of the activity of the single catalyst particles is shown in Fig. 2e, and the position at which the fluorescent intensity is measured is highlighted with a yellow square. The walls of the microfluidic channels are made hydrophobic by flushing the flow channel with a solution of tridecafluoro-1,1,2,2-tetrahydrooctyltrichlorosilane (FOTS). For the oligomerization reaction experiments, flow rates of 5 µL/min were used for the droplet phase and 9 µL/min for the continuous phase, resulting in a total flow rate of 14 µL/min. The length of the fluidic channel is 32.3 cm, and the velocity of the droplets is 8.3 mm/s. The fluorescent intensity measurement point is located 30.7 cm after the droplet generator, resulting in a residence time of 37 s per droplet from the point of generation until the detection spot.

Measurements were performed at 30 °C, 70 °C, and 95 °C to determine the optimum reaction temperature and to ensure that the chemical staining reaction does not occur at room temperature inside the feed syringe. A temperature of 95 °C was selected to avoid the appearance of bubbles from the paraffin oil, as seen in preliminary experiments (which are not shown for the sake of brevity). More details on the precautions taken against bubble formation are described in the Materials and Methods section.

A sieved fraction (38–75 µm) of the catalyst particles from the general size range (50–150 µm) was used to prevent the largest particles from clogging the channel. Additional information on the particle size distribution can be found in Section SI.2. Despite using this sieved fraction of smaller catalyst particles, precautions were taken to keep them suspended and avoid sedimentation (i.e., the particles, which have a skeletal density of 2.4–2.8 g/cm^3^ versus a density of 0.8 g/cm^3^ for the paraffin oil, would sink to the bottom of the syringe and/or capillary). Therefore, a Teflon-coated magnetic stir bar was added inside the syringe to keep the particles suspended. Moreover, a vibrational motor was mounted to the tip of the syringe to shake the tubing and prevent the particles from getting stuck in the glass capillary. Both the magnetic stir bar and the vibrational motor are shown in Section SI.3.

**Fig. 3:**
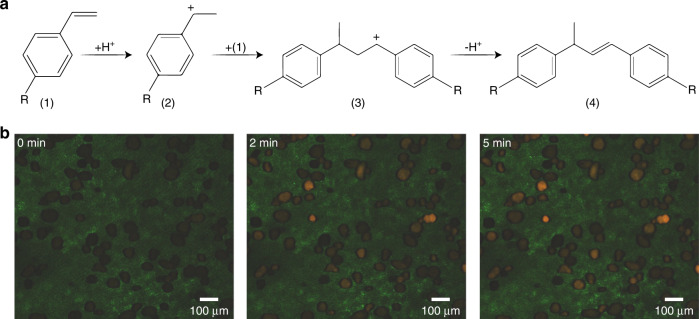
Oligomerization reaction of 4-methoxystyrene catalyzed by FCC ECAT particles. **a** Reaction mechanism for the oligomerization reaction of 4-methoxystyrene catalyzed by the FCC ECAT, and **b** batch test results determined at three time intervals (0, 2, and 5 min) for the probe reaction occurring within the FCC ECAT particles at 100 °C in paraffin oil. Green is the background color, orange/red dots are the more acidic/fresh particles and dark dots are the deactivated particles. The contrast and brightness have been enhanced equally for each image by using Adobe Photoshop for visibility purposes only. Furthermore, all initial microscope settings were equal for all images

A batch test of the reaction was performed in which formation of the fluorescent reaction product via acid-catalyzed oligomerization of 4-methoxystyrene was monitored over 5 min at 100 °C with a Linkam cell. The reaction mechanism for oligomerization of the styrene derivatives, including 4-methoxystyrene, is shown in Fig. 3a^50^. In Fig. 3b, the fluorescence signals generated after 0, 2, and 5 min are shown. It is clear that large amounts of fluorescent product were formed in some particles, while others did not show any fluorescence.

### Reaction temperature determination

**Fig. 4:**
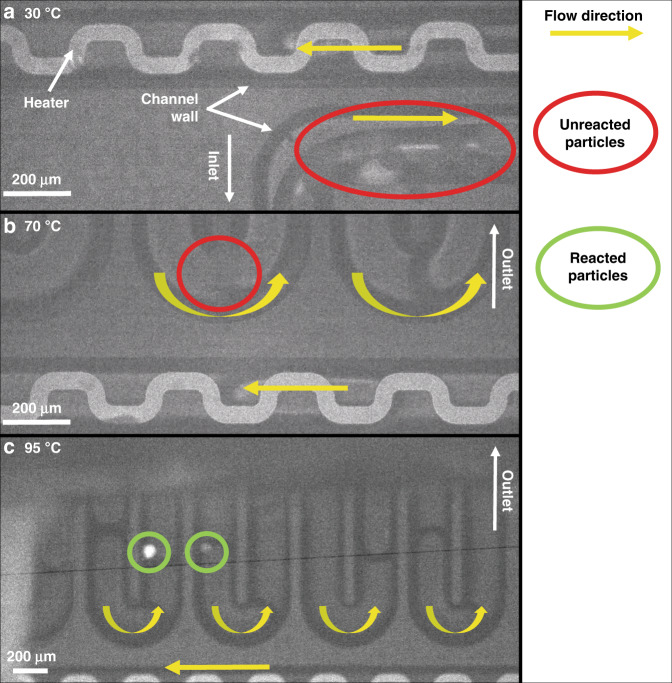
FCC ECAT particles at various temperatures and positions inside the microreactor. FCC ECAT particles observed at various temperatures and positions in the microreactor, with **a** catalyst particles at the inlet that did not react at 30 °C, **b** a catalyst particle in the optical window region near the outlet that had not reacted at 70 °C, and **c** two catalyst particles in the optical window region with different fluorescent intensities after reacting at 95 °C in the microreactor

Figure 4a shows FCC ECAT catalyst particles at the inlet at 30 °C, and it is clear that there is no fluorescent signal, indicating that the reaction did not occur inside the syringe. Furthermore, increasing the temperature to 70 °C did not result in a fluorescent signal near the outlet of the microreactor, as shown in Fig. 4b. It is difficult to see the, but the absence of an interface between the paraffin oil droplet and the continuous phase (i.e., FC-40 oil) indicates the presence of a particle. After increasing the temperature to 95 °C, the catalyst particles showed a fluorescence signal. The videos of the experiments run at 30, 70, and 95 °C can be found in Section SI.5 (Supplementary Videos 1–3). Figure 4c shows two different particles, one with a high-intensity fluorescence signal and the other with a low-intensity fluorescence signal. Because no signal was seen at 70 °C, the chance that a reaction started inside the syringe was negligible, and the observed fluorescence at 95 °C was generated during the residence time of the particle in the microreactor (37 s). Therefore, it was concluded that 95 °C is the optimum temperature for monitoring the reaction in the microreactor. This temperature was used for the experiments described in the next section.

### Measuring and analyzing fluorescent catalyst particles

**Fig. 5:**
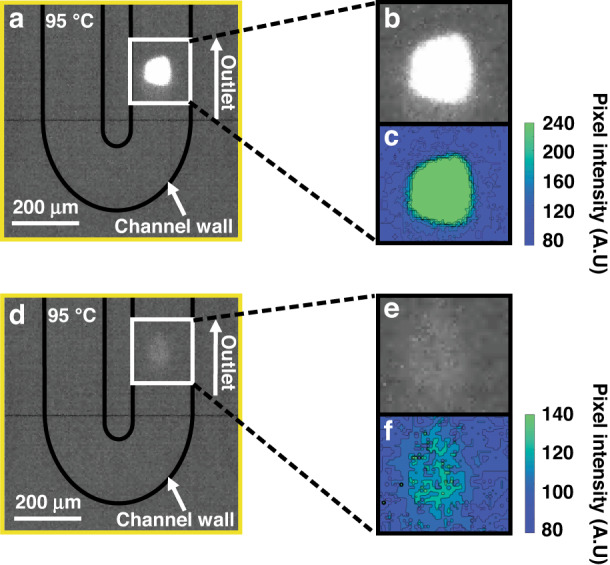
Fluorescence intensity of two different FCC ECAT particles at 95 degrees Celsius inside the microreactor. Fluorescence intensities of two FCC ECAT catalyst particles measured in the detection area of the microreactor at 95 °C; **a** a particle with a high fluorescence intensity and **d** a particle with a lower intensity. **b**, **c**, **e**, and **f** show expanded views and color maps with pixel intensity color bars for **a** and **d**, respectively

**Fig. 6:**
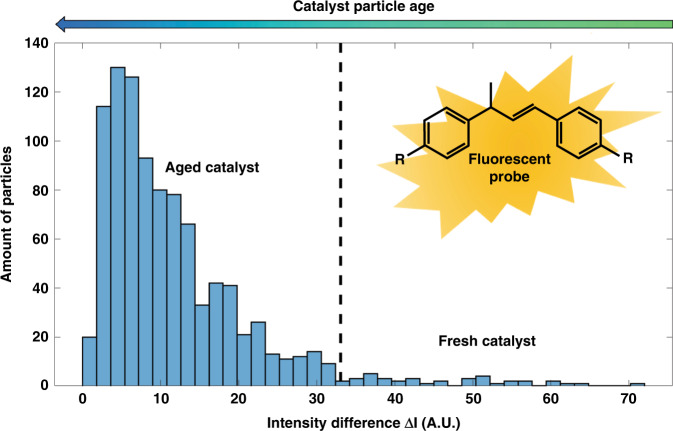
Fluorescence intensity distribution for the analyzed FCC ECAT particles. Histogram showing fluorescence intensity differences for the FCC ECAT catalyst particles on the x-axis and the number of catalyst particles with a particular fluorescence intensity difference on the y-axis

High-throughput fluorescence-based screening was carried out in the microreactor at a temperature of 95 °C. The fluorescence of the FCC ECAT particles was measured in the detection area (indicated by the yellow square in Fig. 2e) with a high-speed camera. With this setup, 967 fluorescent catalyst particles were detected at an average rate of 1 particle per 2.4 s. Analyses of the data from the videos recorded during the experiments was performed with MATLAB. The analyzed videos can be found in Section SI.5 (Supplementary Videos 4–20). The frames containing a fluorescent particle were found with an algorithm that searched the video file for frames containing higher than background fluorescence. After the frames were collected, another MATLAB script was used to find the approximate edges of the particles. Figure 5a shows an example of a frame containing a particle exhibiting high fluorescence intensity, which was found with the MATLAB script. Figure 5b shows an expanded view of this particle, and Fig. 5c shows a converted image with a colormap showing the pixel intensities of the particle. Figure 6d shows a frame, which was detected with the MATLAB script, that contained a particle emitting low-intensity fluorescence that was difficult to distinguish from the background. Figures 5e, f show the expanded view and a colormap of the particle in Fig. 5d, respectively. Figure 5f shows that this particle had a maximum fluorescence intensity of approximately 140, whereas the bright particle in Fig. 5c had its lower intensities at approximately 140. The lowest intensity values for the weakly emissive particles were close to the background intensity, which had an average value of 91. The total range of pixel intensities was 0 to 255, and the maximum intensity found for all particles was 252. This meant that even for the brightest particle, the pixels were not saturated. To determine the emission intensity of a particle, all pixels within the expanded window exhibiting emission intensities above the threshold value of 94 were counted and divided by the total number of pixels found above this threshold value. Figure 6 shows a histogram for the particle intensity distribution.

The fluorescence intensity differences (ΔI) are given on the x-axis, meaning that the set of particles found with intensities slightly above the threshold is placed at 0, and the intensity differences of the other particle sets are shown relative to this lowest set. Furthermore, the fluorescence of the catalyst particles that did not react, as shown for the batch test in Fig. 3 as well as in Fig. 4b (on-chip experiment), was not distinguishable from the background level, so these particles were not detected. Section SI.4 contains more details indicating what this means for the actual acidity distribution for the catalyst particles in the reactor. The histogram in Fig. 6 can be divided into two parts. Most (i.e., 929/967 or 96.1%) of the catalyst particles were aged and already deactivated and showed low-to-moderate fluorescence intensity, whereas a minority (i.e., 28/967 or 3.9%) of the catalyst particles exhibited high fluorescence intensities and are considered to be fresh catalyst particles. These could have been particles that were most recently added to the ECAT mixture or particles that had experienced multiple reactor cycles but still performed as fresh particles. The threshold intensity for classification of these particles was between 30 and 40 on the x-axis in Fig. 6. Due to the direct correlation of measured particle emission intensity with the activity of the particle, the particles on the right-hand side of this threshold were highly active and therefore the so-called particles of interest. The strong fluorescence caused by the available Brønsted acid sites indicated that these catalyst particles were less likely to be deactivated by the harsh reaction conditions experienced in a commercial FCC reactor unit. Assuming that the particle activity distribution for the entire ECAT population showed a normal family (e.g. normal or lognormal) distribution, the 967 on-chip analyzed catalyst particles constituted a representative sample for the reactivity distribution of the entire population. For this many particles, the measured sample was representative of the actual population with a confidence level of 95%. Furthermore, there was an error margin of 3.2%, which indicates the range in which the measured sample may differ from the actual population. The calculations indicating the representativeness of the sample size can be found in Section SI.4.

## Conclusions

We have described a droplet microreactor used for high-throughput screening of individual fluid catalytic cracking (FCC) equilibrium catalyst (ECAT) particles based on their Brønsted acidities. With this high-throughput single particle screening method, a compromise between bulk measurements that average the characteristics of multiple particles and single particle measurements that lack statistical relevance has been developed and demonstrated. The screening was performed in situ at elevated temperatures up to 95 °C by using a fluorescent reaction product as an indicator of catalyst acidity. We oligomerized 4-methoxystyrene with calcined FCC ECAT particles located inside paraffin oil droplets. In total, ~1000 FCC catalyst particles were detected with an average rate of 1 particle per 2.4 s. This provided statistically relevant results for the single catalyst particle study, given that this number of detected FCC particles was representative of the entire population with a confidence level of 95%. Because the microreactor contained integrated heaters and temperature sensors, the reaction conditions could be tuned, and the optimum reaction conditions were found. The highly active particles generated strong fluorescence signals after a residence time of 37 s at 95 °C. Videos of the fluorescent catalyst particles passing the detection spot were recorded and analyzed with MATLAB. The data showed a clear distinction between the majority (≈96.1%) of the bulk catalyst particles that exhibited low-to-moderate fluorescence intensities and a minority (≈3.9%) of highly active particles showing strong fluorescence. This screening platform can provide valuable information on the age distribution of the catalyst particle mixture and the overall reactor acidity while also providing information on the acidity of individual catalyst particles. We envision that this technique will be used to study the influence of reactor operating conditions on the catalyst particle population or to study the effects of synthesis parameters on the performance of the catalyst particles. Future work could combine on-chip acidity screening with the previously reported fluorescence-based dielectrophoretic sorting of catalyst particles based on their acidity^10^.

## Materials and methods

### Platinum temperature sensor characterization and calibration

Characterization and calibration of the Pt temperature sensors were achieved by mounting the microreactor on a printed circuit board (PCB) and connecting the electrodes to the PCB, after which the microreactor was controlled with external connectors. The microreactor was fully immersed in a beaker of oil standing on a hotplate. A thermocouple connected to the hotplate was used as a reference thermometer and measured the temperature of the oil. A magnetic stirrer was added to increase the temperature uniformity of the oil. The hotplate was cycled from 30 °C to 150 °C and back to 30 °C in steps of 10 °C to rule out hysteresis effects. In addition, the resistances of the temperature sensors in the microreactor were measured. The signals from the on-chip temperature sensors (TS1 and TS2) were measured by applying a current with an LM317TG voltage regulator as the current source and subsequently measuring the voltage. The voltage across the resistor was first amplified with an AD620ANZ instrumentation amplifier. The output signal was recorded with a National Instruments (NI) myRio data acquisition board, which was connected to LabVIEW software on a computer. The electronic design can be found in Section SI.5. With the obtained resistance versus temperature curve, the RTD was calibrated and used as a temperature sensor for feedback and control during other experiments with the droplet microreactor.

### Microreactor setup for performing chemical staining reactions

Droplets were created inside the microreactor with a Nemesys (Cetoni GmbH, Korbussion, Germany) syringe pump fitted with a 1000 µL glass Hamilton syringe for the droplet phase (paraffin oil with particles and reactant) and a 500 µL glass Hamilton syringe for the continuous phase (fluorinated FC-40 oil (Fluorinert 3 M)). The pump was controlled with Nemesys software, and the syringes were connected to the microreactor with fused silica tubing (Polymicro Technologies, ID = 250 µm, OD = 360 µm) and connectors (NanoPort ferrule: N-123-03, NanoPort nut: F-123H, MicroTight connector: P-882) from Idex-HS. The microfluidic channel walls were made hydrophobic by flushing the flow channel with a solution of 5.55 µL of tridecafluoro-1,1,2,2-tetrahydrooctyltrichlorosilane (FOTS, Sigma-Aldrich) in 1.5 mL of FC-40 oil. The microreactor was flushed for 45 min with the FOTS/FC-40 oil mixture and then for 45 min with only the FC-40 oil. The vibrational motor used to preclude particle sedimentation was from Mouser Electronics.

### Catalyst and reactant preparations

Bulk measurements with a Nikon Eclipse 90i confocal fluorescence microscope with an A1R scan head (10x objective) equipped with a 488 nm argon ion liquid state Melles Griot laser (40 mW) showed that the reaction product had an emission maximum at 600 nm when excited at 488 nm. An FCC ECAT sample was calcined for 5 h at 600 °C (1 °C/min to 120 °C, held for 1 h, then 5 °C/min) to remove all residual coke. A sieved 38–75 µm fraction was used for further experiments. Six milligrams of the calcined ECAT particles was impregnated with 10 µL of 4-methoxystyrene (Sigma-Aldrich, 97%). Two milliliters of the paraffin oil (Sigma-Aldrich, analytical grade) was added to the reactant/particle mixture, and after shaking to create a suspension, the oil/reactant/particle mixture was put into a 1000 µL glass Hamilton syringe. During preliminary experiments with the microreactor, gas bubbles from the untreated paraffin oil appeared when the temperature reached 90 °C. It was expected that these gas bubbles were caused by volatile compounds in the paraffin oil. Therefore, prior to use in the microreactor, the paraffin oil was heated to 100 °C for 5 h to remove volatile compounds. After this treatment, no bubbles appeared when the paraffin oil was heated to 95 °C in the microreactor.

### Fluorescence microscopy setup

A Leica DMi 5000M inverse microscope with a 10x objective equipped with a Hg lamp and a BGR filter cube was used for the fluorescence measurements. The BGR filter cube has three excitation bands (420 nm ± 30 nm, 495 nm ± 15 nm, 570 nm ± 20 nm) and three emission bands (465 nm ± 20 nm, 530 nm ± 30 nm, 640 nm ± 40 nm). A Hamamatsu Orca Flash4.0 V2 camera was used to capture the fluorescence images. The exposure time of the camera during filming was set to 1 ms, and the frame rate was set to 200 fps.

## Supplementary information

SI for Droplet Microreactor for the High-Throughput Fluorescence-Based Detection of Single Catalyst Particle Activity_revision_no_markup
