# Supplementary material for: Droplet microreactor for high-throughput fluorescence-based measurements of single catalyst particle acidity
Source: Microsyst Nanoeng. 2023 Mar 30;9:39. doi: 10.1038/s41378-023-00495-2 (PMC10060574; doi:10.1038/s41378-023-00495-2)
Supplement: Supplementary file 1 — SI for Droplet Microreactor for the High-Throughput Fluorescence-Based Detection of Single Catalyst Particle Activity_revision_no_markup [file 41378_2023_495_MOESM1_ESM.docx]

Supplementary Information for:

Droplet Microreactor for High-Throughput Fluorescence-Based Measurements of Single Catalyst Particle Acidity

Jeroen C. Vollenbroek^1†^, Anne-Eva Nieuwelink^2†^, Johan G. Bomer^1^, Roald M. Tiggelaar^3^, Albert van den Berg^1^, Bert M. Weckhuysen^2^, Mathieu Odijk^1^

^1^ BIOS Lab on a Chip Group, MESA+ Institute, University of Twente, Hallenweg 15, 7522 NH Enschede, The Netherlands

^2^ Inorganic Chemistry and Catalysis, Debye Institute for Nanomaterials Science, Utrecht University, Universiteitsweg 99, 3584 CG Utrecht, The Netherlands

^3^ NanoLab cleanroom, MESA+ Institute, University of Twente, Hallenweg 15, 7522 NH Enschede, The Netherlands

# SI.1 Microreactor fabrication

Figure S.1 shows an overview of the executed fabrication steps. The microreactor is a stack of a 525 µm thick P-type <100> silicon substrate, and a 500 µm thick Mempax glass substrate. A three-mask process is used to realize the desired features both in and on the substrates, as shown in figure S.1. Using Buffered HF (BHF) as a wet etchant, the pattern for the Pt structures is etched 200 nm deep into the glass substrate. This process is directly followed by the deposition of a 10 nm thick tantalum adhesion layer and a 190 nm thick platinum layer, thus the heaters and temperature sensors are embedded into the glass substrate. Next, the complete glass substrate containing the Pt structures is covered with 1 µm SiO_2_ ­deposited with Plasma Enhanced Chemical Vapour Deposition (PECVD). The SiO_2_ is then selectively removed from the contact electrodes, using standard photolithography and Reactive Ion Etching (RIE). The channels are etched 150 µm deep into the silicon substrate using the Bosch process for Deep Reactive Ion Etching (DRIE). Fluidic and electronic accesses are etched from the backside of the silicon substrate using DRIE. This ensures accessibility to both the electrodes embedded in glass, and the flow channels after the substrates are bonded together. The silicon substrate is dry oxidized, to create a 200 nm thin insulating SiO_2_ film on the walls of the channel. After completion of all the process steps, the silicon and glass substrates are anodically bonded, and the individual microreactors are created by means of dicing.

| 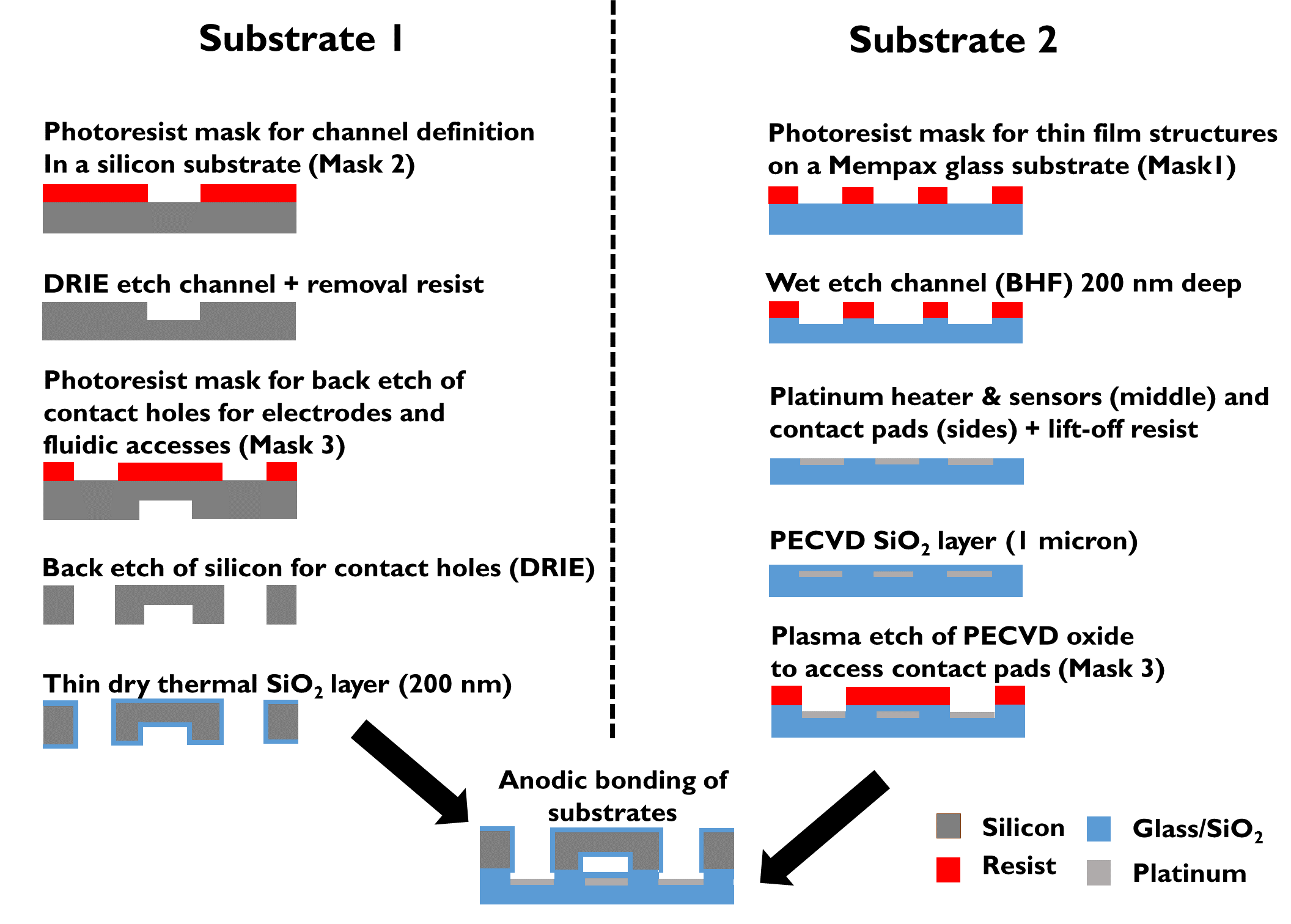 |
| --- |
| Figure S.1: Schematic cross-sectional representation of the various steps used to fabricate the droplet microreactor. © 2017 IEEE. Reprinted, with permission, from J.C. Vollenbroek *et al.*^1^ |

The fabrication result is shown in figure S.2 where the silicon/glass droplet microreactor is shown with the designed features such as, temperature sensors, droplet generator, inlets, heaters, optical window, and outlet. Furthermore a microscopy image of TS2 and the heater structure (H1 – H3) are shown in figure S.2

| 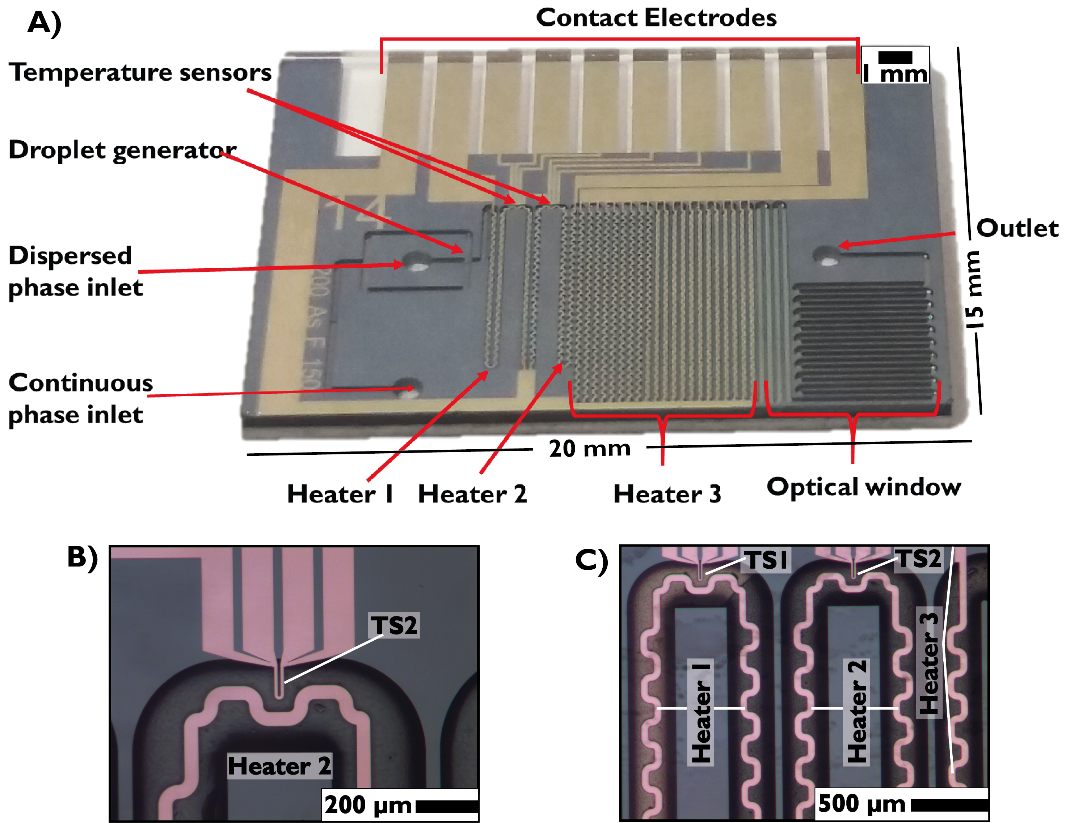 |
| --- |
| Figure S.2: A) Images of the fabricated silicon/glass droplet microreactor, showing the temperature sensors, droplet generator, inlets, heaters, optical window and outlet. Optical microscopy images of the temperature sensors (TS1 and TS2) and the heaters are shown in B) and C), respectively. |

# SI.2: Particle size distribution

FCC ECAT particles size distribution of a 38 - 75 $\mu m$ sieved fraction was determined by analyzing microscopy images using ImageJ. The area of each particle was determined. The catalyst particles are not spherical and can have quite oblong shapes. However, the diameter of the particle for the distribution was calculated as if they were perfectly spherical. Figure S.3 shows the histogram of the catalyst particle size distribution for a total number of 310 particles. The particles with a diameter above 75 $\mu m$ are most likely caused by the non-spherical shape, where they fit through the filter in one direction, but not the other. A boxplot of the distribution is shown in figure S.4 with a median particle size of ~60 $\mu m$.

| 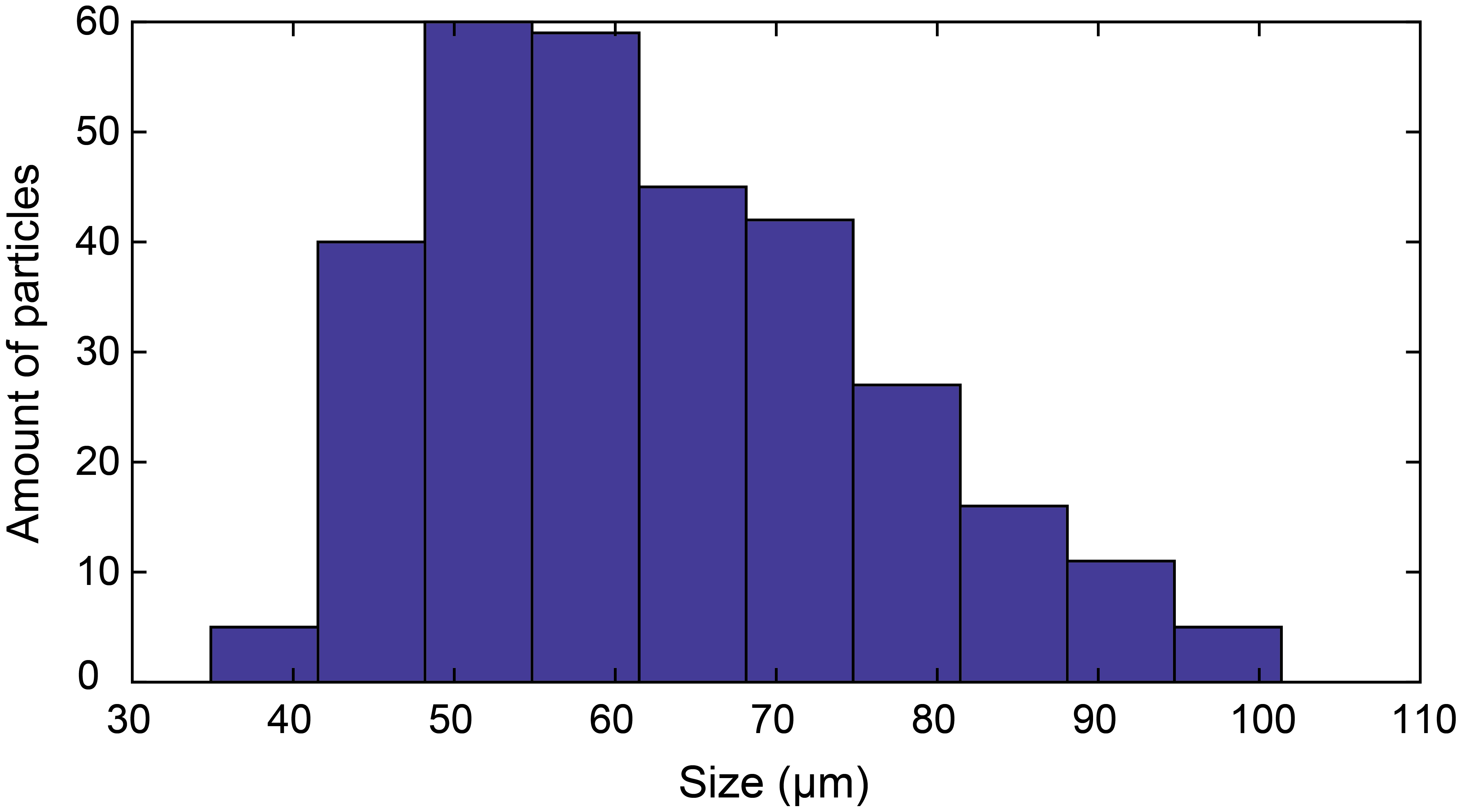 |
| --- |
| Figure S.3: Histogram showing the FCC ECAT particle size distribution of the 38 - 75 μm sieved fraction for a total number of 310 particles. |

| 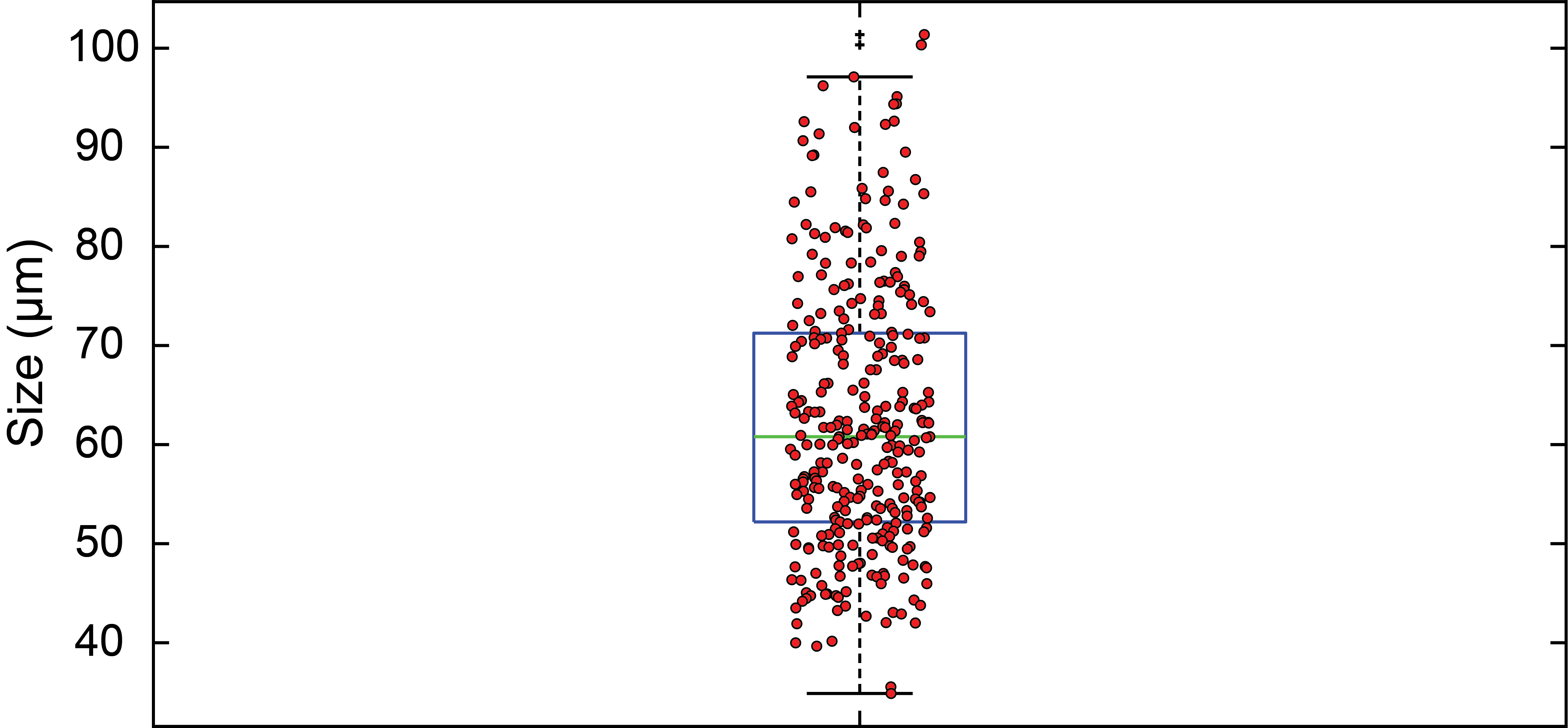 |
| --- |
| Figure S.4: Boxplot of the measured catalyst particle sizes for 310 particles with a median particle size of 60 μm. |

# SI.3: Particle sedimentation precautions

A schematic representation of the magnetic stir bar and vibrational motor used to prevent particles sedimentation in the syringe and tubing is shown in figure S.5, while
figure S.6 shows the actual setup, where as an extra precaution against particle sedimentation the syringes are positioned vertically.

| 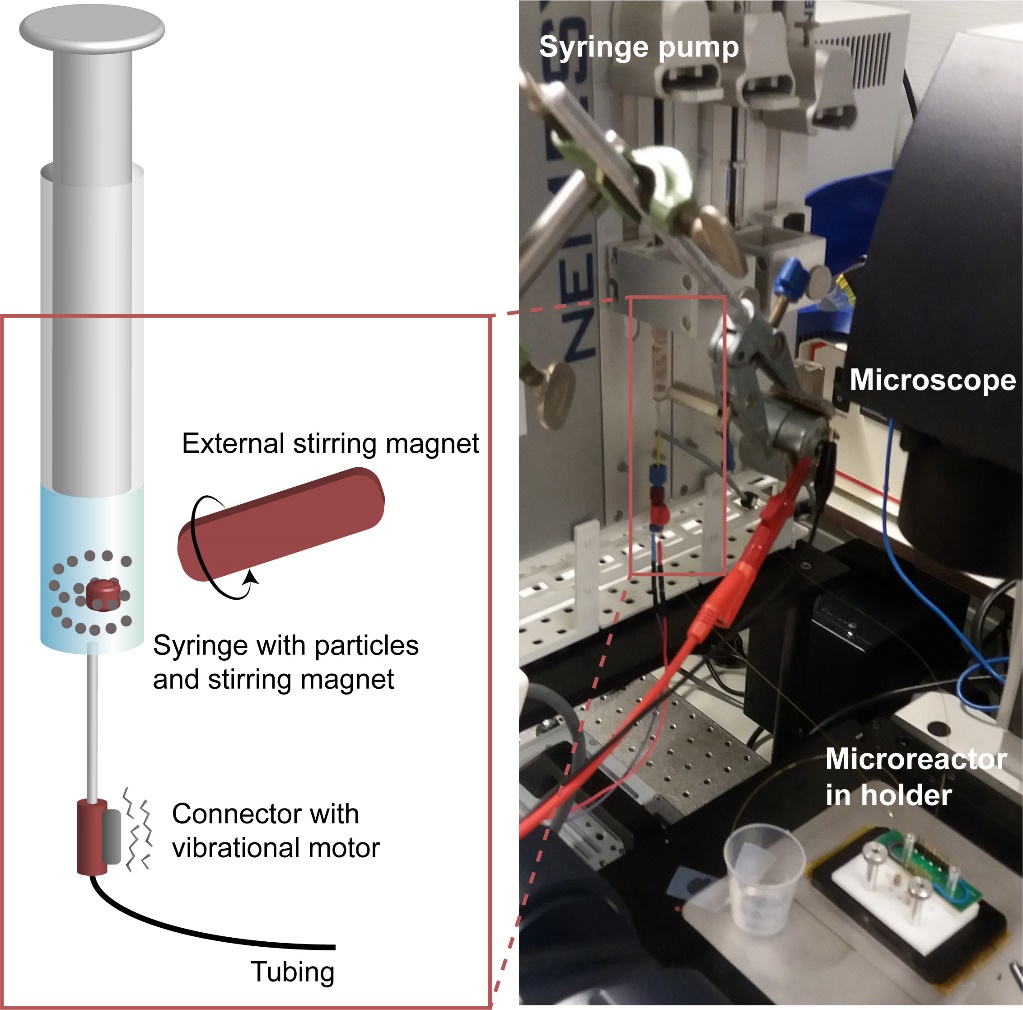 | 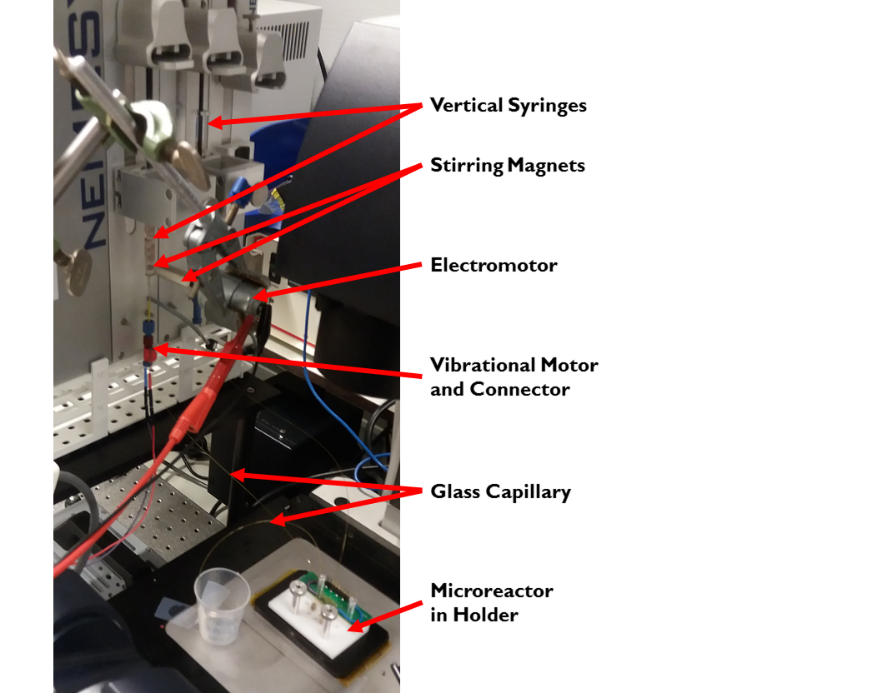 |
| --- | --- |
| Figure S.5: Precautions to keep the ECAT FCC particles in suspension and prevent them from sedimentation. The small stirring magnet inside the syringe, as well as the electromotor that is driving it, are shown. The vibrational motor added to the tip of the syringe is also highlighted. | Figure S.6: Setup used during the activity staining experiments showing the stirring precautions (magnets, vibrational motor and vertical syringes), glass capillaries, and the holder for the microreactor. |

# SI.4: Sample size calculation

The obtained sample of 967 particles was tested with Matlab using the Anderson-Darling test against the null hypothesis that the sample is from a population with a lognormal distribution at a 5% significance level. The test failed to reject the null hypothesis with p = 0.3846, indicating that the sample distribution is part of a lognormal distributed population. As shown in figure S.7 the obtained sample histogram could be fitted to a lognormal distribution using Matlab. Although it is not possible to measure particles that exhibit a fluorescence intensity below the background signal, these particles would show up on the left side of the histogram. It is not expected that the missed particles would skew the distribution in such a way that it is not part of the normal family (it is always possible to transform it to a normal distribution). A guide to the eye for the expected particle distribution inside the reactor that compensates for the non-detected particles is added to figure S.7.

| 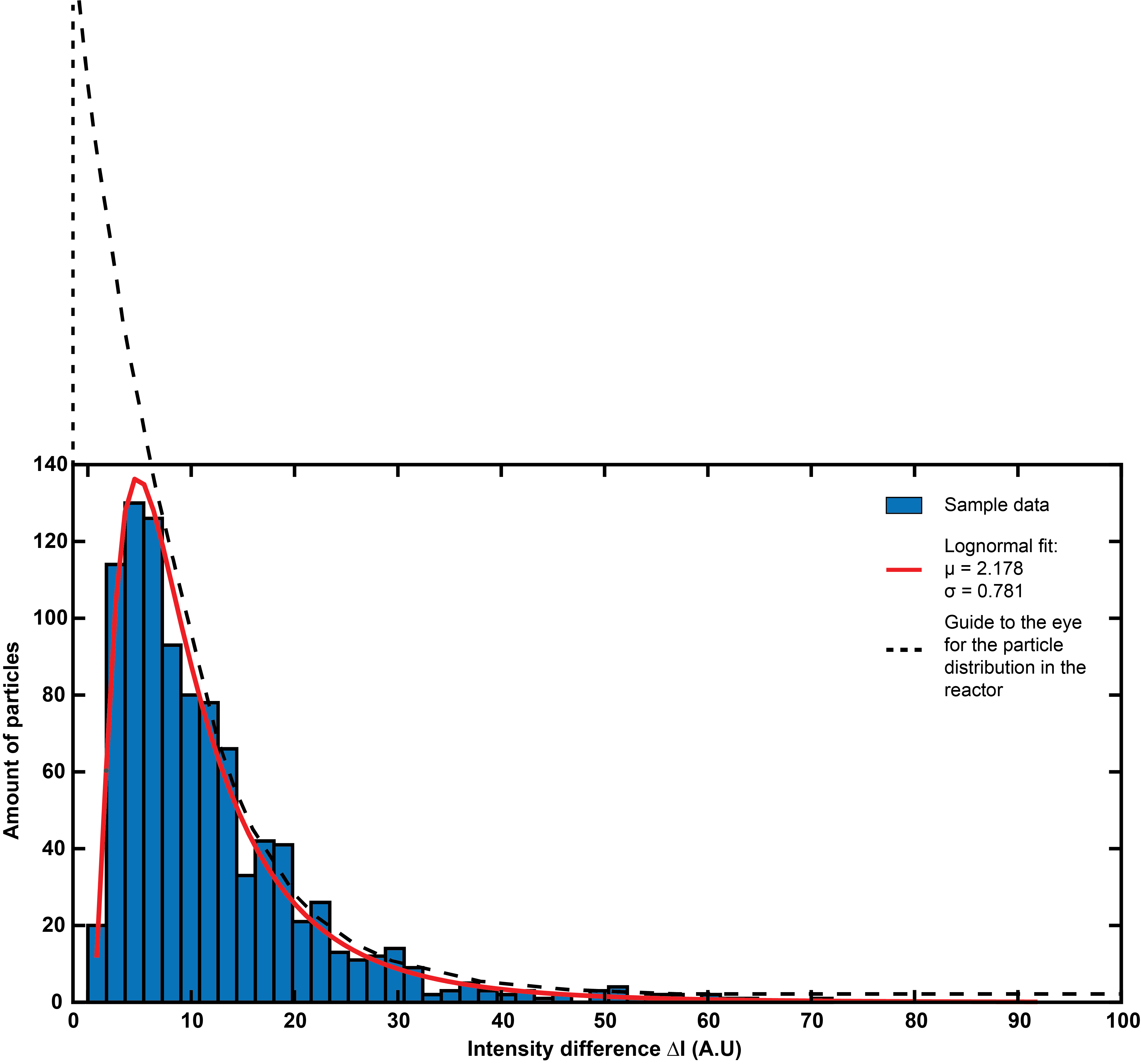 |
| --- |
| Figure S.7: Sample data distribution (blue bars) and lognormal fit with the mean (µ = 2.178) and the standard devation (σ = 0.781) of the logarithmic values. The dotted line represents a guide to the eye for the particle acidity distribution in the reactor compensated for the non-detected particles. |

Given that the sample is part of the normal family and that the entire population is large (there are more than 1 billion particles in a reactor), Cochran’s sample size formula can be used as shown in equation (eq. S.1)^2^.

$sample size= \frac{z^{2}p\left( 1-p \right)}{e^{2}}$ (eq. S.1)

Where z is related to a desired confidence level (chance that the sample population represents the entire population) that can be found in table S.1 below for various confidence levels, p is the estimated proportion of the population which has the attribute that is tested for, and e is the desired margin of error. Typically p is set to 0.5 if the proportion is unsure. A proportion of 0.5 results in the largest estimated sample size^2^ with respect to other p values. Given the 967 particles in the measured sample, an error margin of 3.15% at a confidence level of 95% is calculated. Typically an error margin of 5% is acceptable in industry for a confidence level of 95%.^2^

| Table S.1: z-value related to desired confidence level. Table taken from^3^ | |
| --- | --- |
| **Confidence level interval** | **z-score** |
| 90% | 1.645 |
| 95% | 1.96 |
| 99% | 2.576 |

# SI.4: Electronic design, read-out and calibration

In this section the schematics of the temperature sensor read-out circuit (figure S.8) and heater control circuit (figure S.9) are explained. This information can also be found in.^4^ In the sensor read-out structure, the LM317TG is used as a current source. The formula for the output current is given by equation (eq. S.2).

$I_{\mathrm{out}}=\frac{1.25}{R_{1}}$ (eq. S.2)

$I_{\mathrm{out}}$ is chosen to be 2 mA as to not heat up the structure by this bias current. This makes $R_{1}$ 625 $\Omega$. The current through the sensor causes a voltage drop across the resistors. This voltage is amplified using an AD620ANZ instrumentation amplifier. The formula used for calculating the gain of this amplifier is given in equation (eq. S.3).

$Gain=\frac{49.4}{R_{Gain}}+1$ (eq. S.3)

The gain is chosen in such a way that the voltage across at the output of the amplifier would be 5V when the resistor has a temperature of 250 $^{\circ}C$. This was done in order to have enough gain in the desired temperature range, but to have some margin with the upper limit of the 12 bit ADC of the myRio. These considerations result in a gain of 82, making $R_{\mathrm{gain}}$ 610 $\Omega$. The 12 bit ADC and the choices for the gain of the instrumentation amplifier result in a resolution of 0.15 $^{\circ}C$ for the complete sensor circuitry.

The microreactor is positioned into a plastic (black Delrin) chipholder, chosen for its low thermal conductivity. Electrical conducting pins are present in the holder which can contact the electrode pads on the microreactor and allow further interfacing with the myRIO. For control of the heaters a Proportional Integral (PI) controller is made in Labview and executed on the myRIO. A PI control loop compares the measured temperature to the setpoint (desired temperature). Based on the difference or error between these two values, more or less power is dissipated. The Proportional factor is the gain with which the controller tries to reach the setpoint and the Integral value takes errors of the past into account, adjusting the gain based on the information. The PI controls the duty cycle of a 1 kHz square wave put onto the gate of a NMOS transistor. The NMOS acts as a switch between a power source, a PMOS, and the heaters. By varying the duty cycle, the power dissipated in the heaters can be controlled. The temperature sensors in the microreactor provide the feedback temperature for the control loop. The schematic for the control circuit is shown in figure S.8. The chipholder, myRio, and a PCB with the control circuit is shown in figure S.9.

| 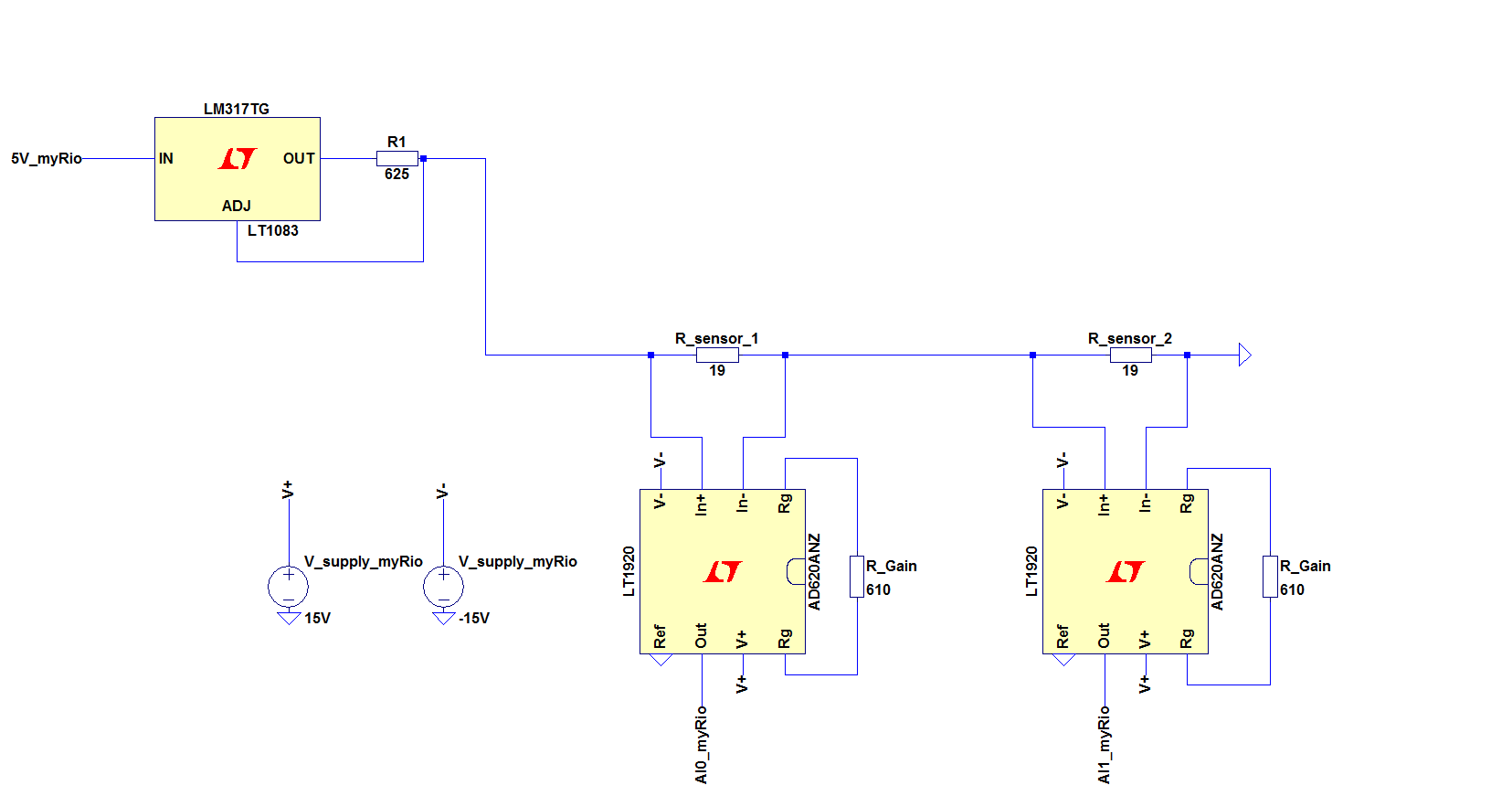 |
| --- |
| Figure S.8: Electrical read-out circuit used in amplification and measurement of the voltage across the temperature sensor structures TS1 and TS2. |

| 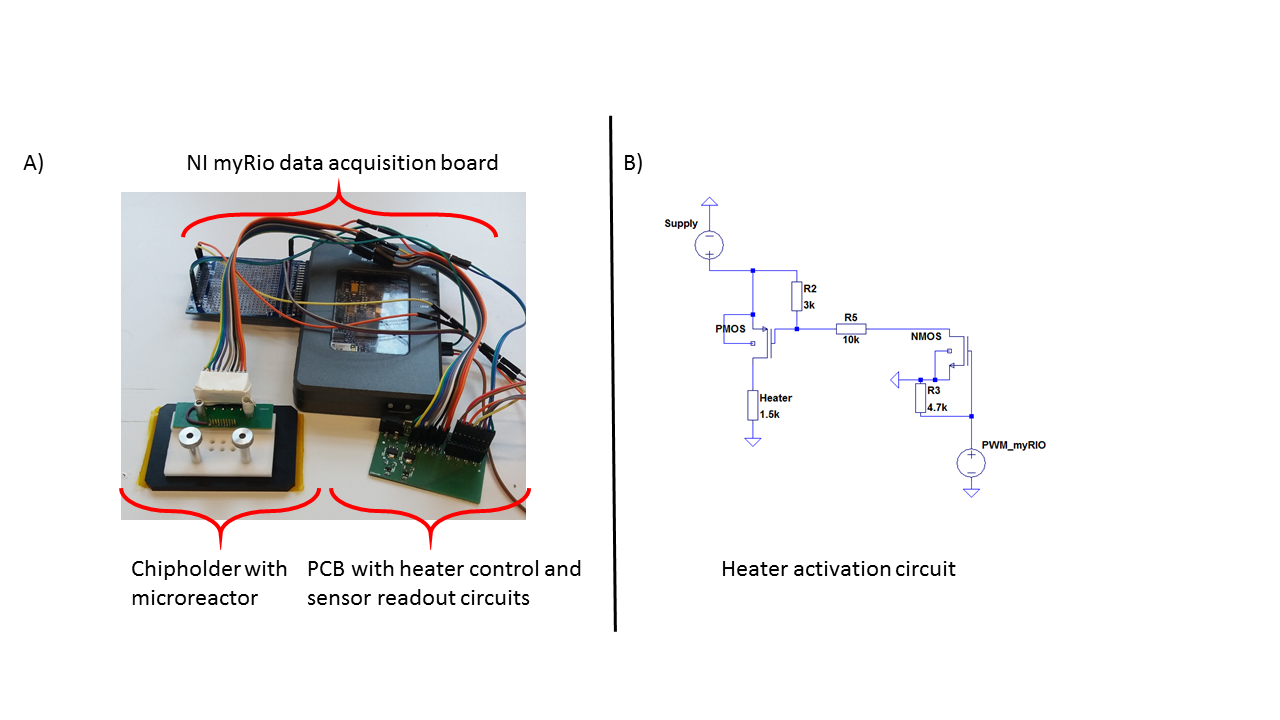 |
| --- |
| Figure S.9: A) the chipholder, NI myRio and PCB with the control electronics used for the read-out and control of the heaters and temperature sensors on the micoreactor. B) Part of the activation circuit on the PCB used for the on-chip heating. |

## Temperature sensor calibration for TS1 and TS2

The temperature characteristics of TS1 and TS2 are given in this section. The characterization is done with the oil immersion method. Figures S.10 and S.11 show the calibration graphs that were obtained for TS1 and TS2. TS1 and TS2 both have a sensitivity of 0.0476 Ω°C^-1^, and an offset of 18.311Ω for TS1 and 18.294Ω for TS2. The measure of linearity, $R^{2},$ is 0.99899 and 0.9989 for TS1 and TS2, respectively.

| 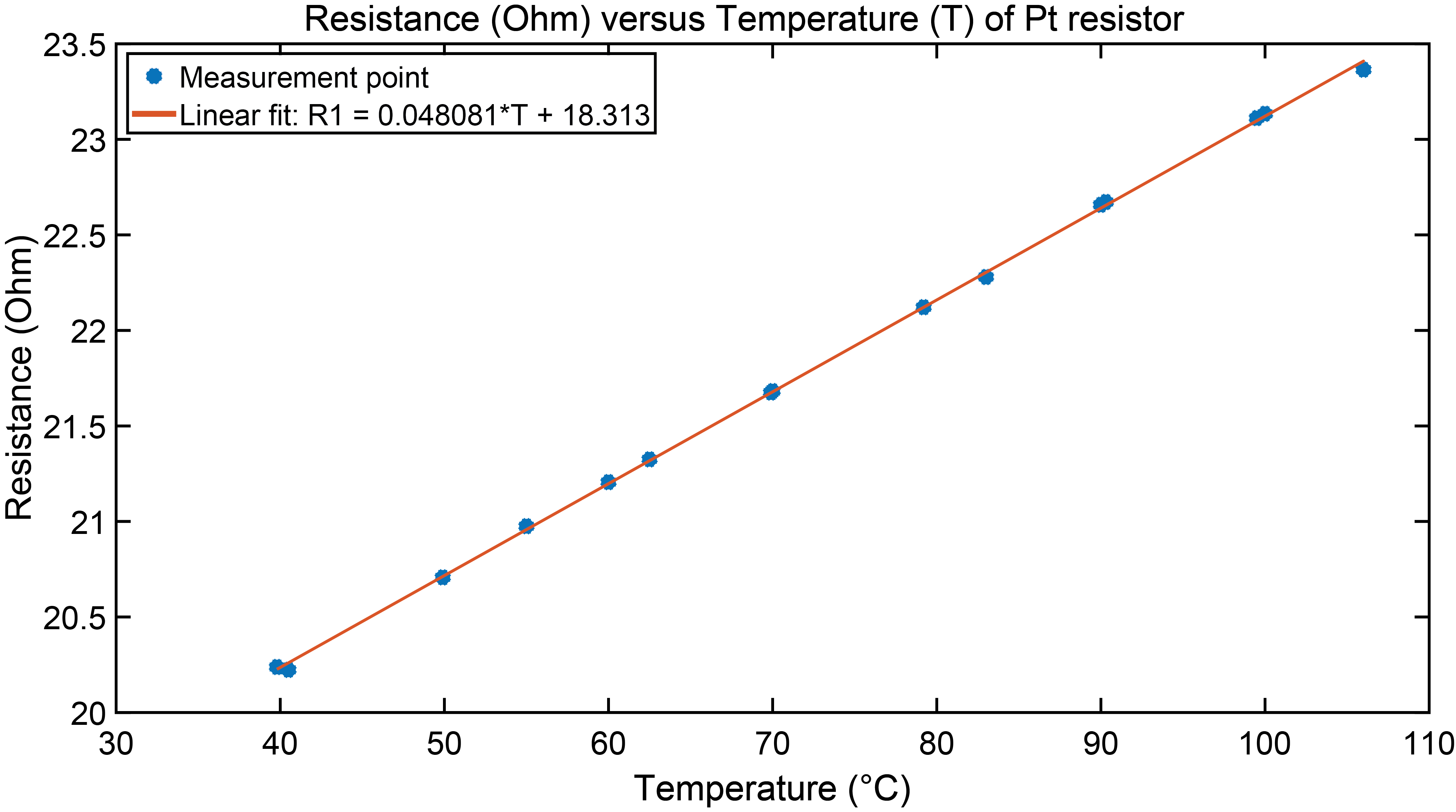 |
| --- |
| Figure S.10: Calibration curve of the temperature sensor TS1 showing the resistance as a function of the temperature. |
| 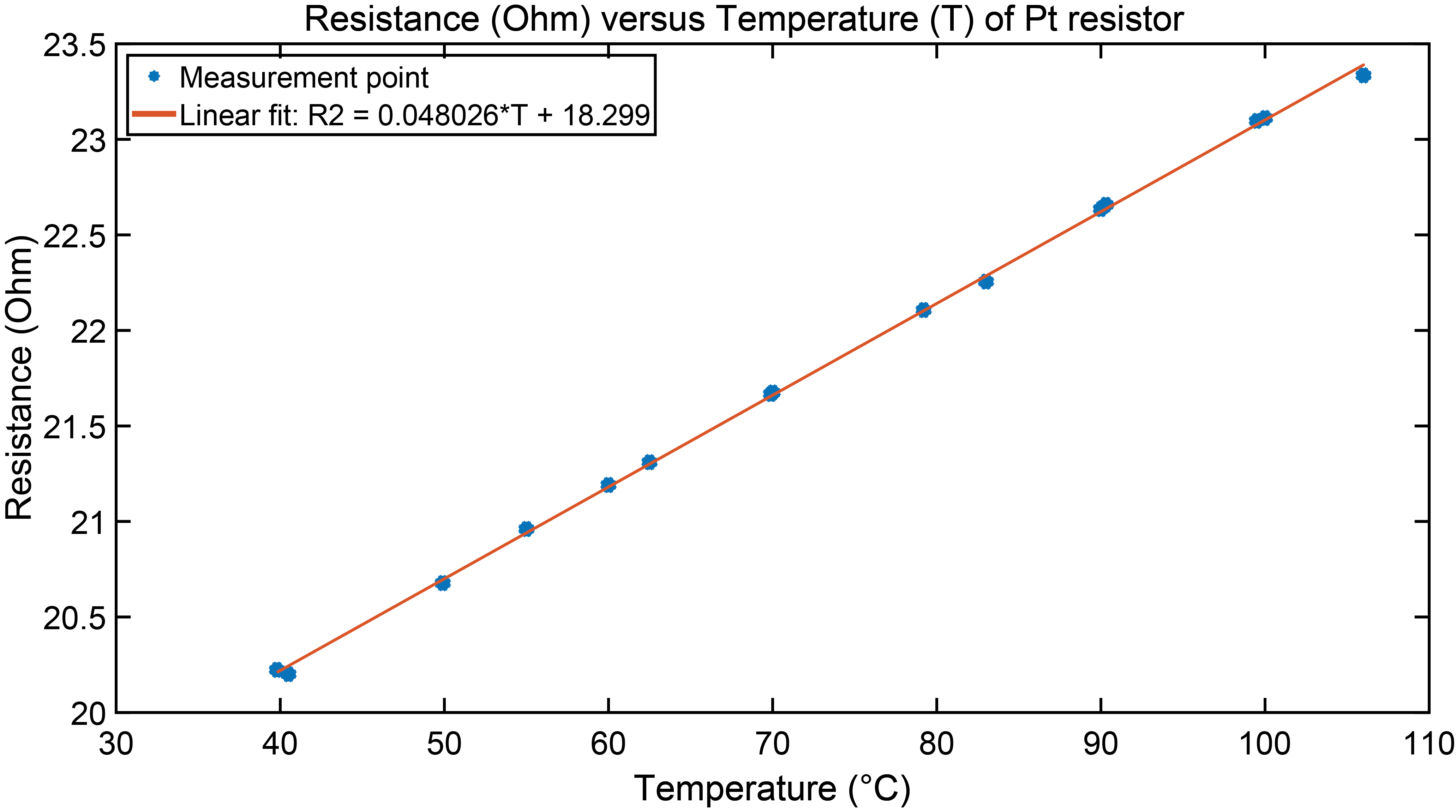 |
| Figure S.11: Calibration curve of the temperature sensor TS2 showing the resistance as a function of the temperature. |

## Heater control measurements

Figures S.12 and S.13 show the temperature readout (in blue) of both sensors (TS1 and TS2) and the corresponding duty cycle (in red) applied to the gate of the NMOS of heaters H1 and H2, for a transient response from 50 $^{\circ}$C to 75 $^{\circ}$C. The duty cycle increases immediately, resulting in an increased power dissipation, and therefore increased temperature of the heater. The response time of the system before it reaches the setpoint is approximately 1.5 seconds, after which the temperature oscillates around the setpoint with a standard deviation of 2.6 $^{\circ}C.$

| 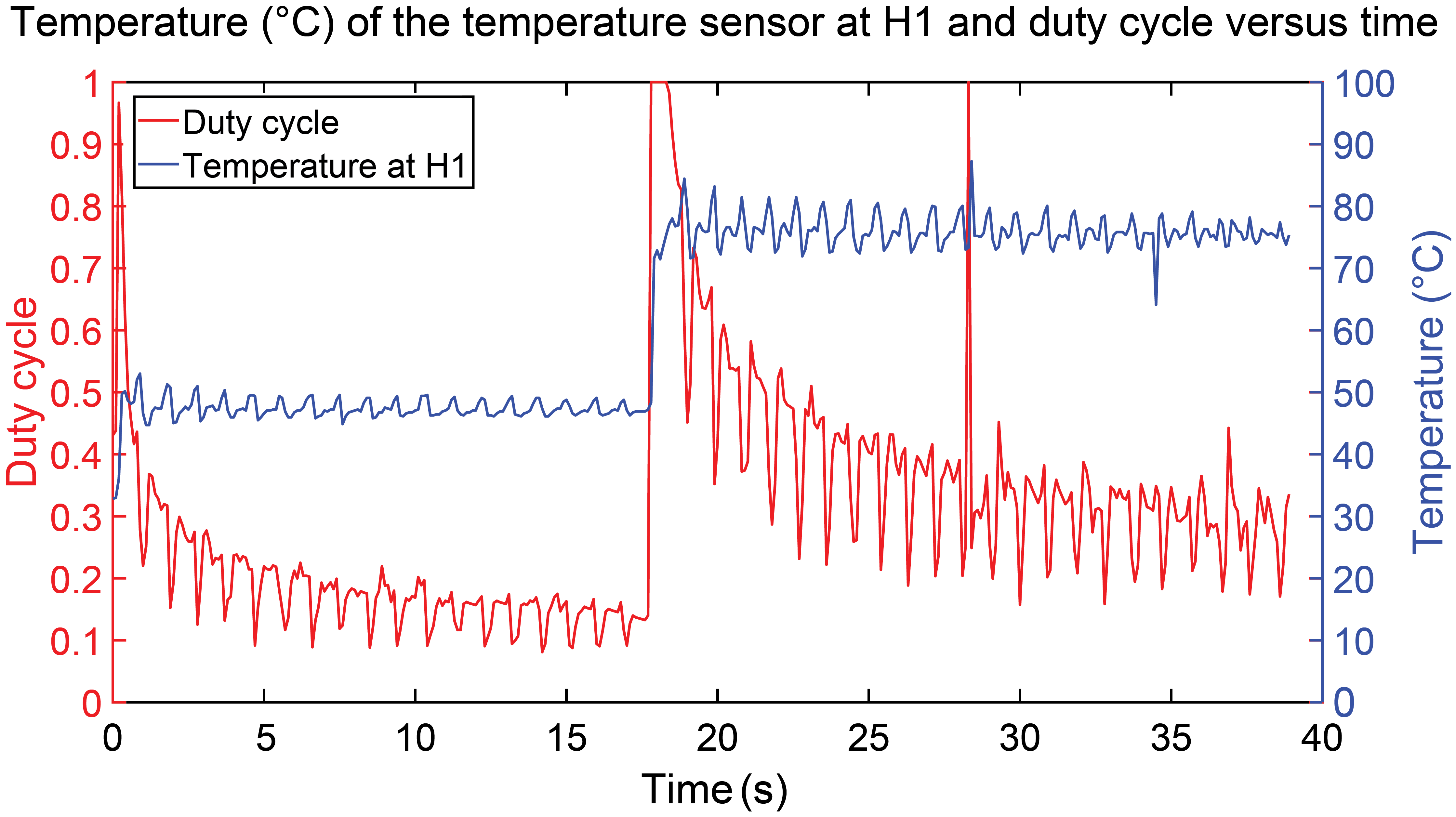 |
| --- |
| Figure S.12: Step response of the control loop showing the temperature increase of heater H1 and the duty cycle controlling the power dissipation. |
| 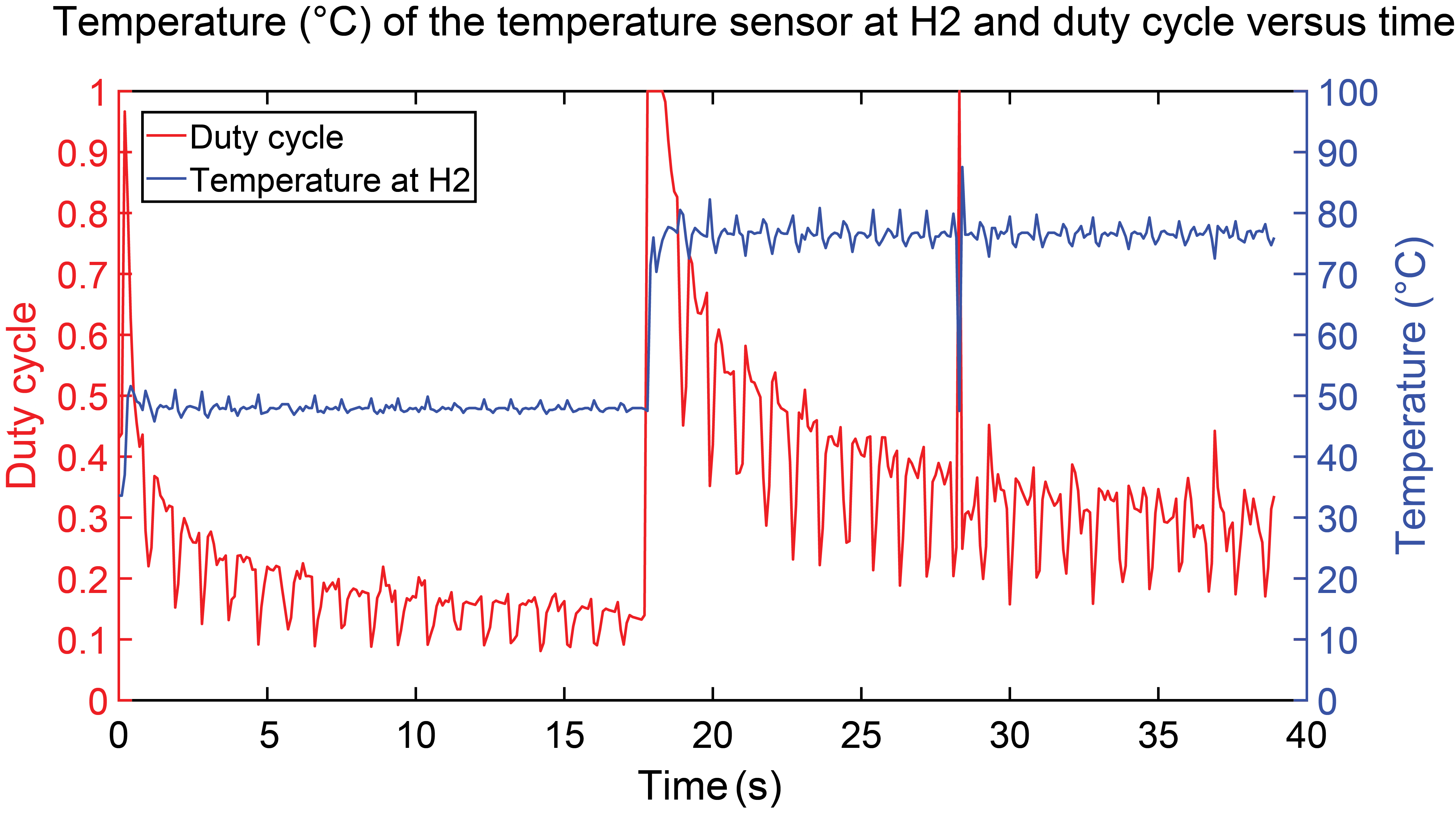 |
| Figure S.13: Step response of the control loop showing the temperature increase of heater H2 and the duty cycle controlling the power dissipation. |

# SI.5 List of videos used in the data analysis

Due to the size of the videos and the upload limit, they can (for now) be found via this link: <https://surfdrive.surf.nl/files/index.php/s/QtC5KlshIQSvBgR>

Supplementary Video 1 to 3 show the experimental videos belonging to Figure 5 from the manuscript, where different reaction temperatures were screened.

Supplementary Video 1: Unreacted (non-fluorescent) particles at the inlet at 30 degrees Celsius.

Supplementary Video 2: Unreacted (non-fluorescent) particles at the outlet at 70 degrees Celsius.

Supplementary Video 3: Fluorescent product in particles at the outlet at 95 degrees Celsius.

Supplementary Video 4 – 20 are the videos used to find and analyze the fluorescent particles that are then processed and put into the histogram in Figure 7 in the main manuscript. Videos are saved at 25 fps, but are recorded at 200 fps.

## References

1. Vollenbroek, J. C. *et al.* Design and characterization of a microreactor for monodisperse catalytic droplet generation at both elevated temperatures and pressures. *2017 IEEE 12th Int. Conf. Nano/Micro Eng. Mol. Syst. NEMS 2017* 746–751 (2017). doi:10.1109/NEMS.2017.8017127

2. Statistics How To. Available at: https://www.statisticshowto.datasciencecentral.com/probability-and-statistics/find-sample-size/. (Accessed: 30th March 2020)

3. Sullivan, L. Confidence intervals. Available at: http://sphweb.bumc.bu.edu/otlt/MPH-Modules/BS/BS704_Confidence_Intervals/BS704_Confidence_Intervals_print.html. (Accessed: 20th March 2020)

4. M.W. van der Helm, V. Papadimitriou, J.C. Vollenbroek, F.T.G. van den Brink (2017). Lab on a Chip: miniaturized devices for chemical and biological analysis. *De Vonk (periodical of the E.T.S.V. Scintilla, University of Twente),* 35(3),6-13.
